# Probing Neuro-Endocrine Interactions Through Remote Magnetothermal Adrenal Stimulation

**DOI:** 10.3389/fnins.2022.901108

**Published:** 2022-06-23

**Authors:** Lisa Y. Maeng, Dekel Rosenfeld, Gregory J. Simandl, Florian Koehler, Alexander W. Senko, Junsang Moon, Georgios Varnavides, Maria F. Murillo, Adriano E. Reimer, Aaron Wald, Polina Anikeeva, Alik S. Widge

**Affiliations:** ^1^Department of Psychiatry, Massachusetts General Hospital and Harvard Medical School, Charlestown, MA, United States; ^2^Research Laboratory of Electronics and McGovern Institute for Brain Research, Massachusetts Institute of Technology, Cambridge, MA, United States; ^3^Department of Psychiatry and Behavioral Sciences, University of Minnesota, Minneapolis, MN, United States; ^4^Department of Materials Science and Engineering, Massachusetts Institute of Technology, Cambridge, MA, United States; ^5^Department of Brain and Cognitive Sciences, Massachusetts Institute of Technology, Cambridge, MA, United States; ^6^Department of Electrical Engineering and Computer Science, Massachusetts Institute of Technology, Cambridge, MA, United States

**Keywords:** epinephrine, corticosterone, adrenal gland, neuromodulation, hormones, magnetic nanoparticles

## Abstract

Exposure to stressful or traumatic stimuli may alter hypothalamic-pituitary-adrenal (HPA) axis and sympathoadrenal-medullary (SAM) reactivity. This altered reactivity may be a component or cause of mental illnesses. Dissecting these mechanisms requires tools to reliably probe HPA and SAM function, particularly the adrenal component, with temporal precision. We previously demonstrated magnetic nanoparticle (MNP) technology to remotely trigger adrenal hormone release by activating thermally sensitive ion channels. Here, we applied adrenal magnetothermal stimulation to probe stress-induced HPA axis and SAM changes. MNP and control nanoparticles were injected into the adrenal glands of outbred rats subjected to a tone-shock conditioning/extinction/recall paradigm. We measured MNP-triggered adrenal release before and after conditioning through physiologic (heart rate) and serum (epinephrine, corticosterone) markers. Aversive conditioning altered adrenal function, reducing corticosterone and blunting heart rate increases post-conditioning. MNP-based organ stimulation provides a novel approach to probing the function of SAM, HPA, and other neuro-endocrine axes and could help elucidate changes across stress and disease models.

## Introduction

Multiple psychiatric disorders, particularly stress and trauma-related conditions, are associated with dysregulation in the hypothalamic-pituitary-adrenal (HPA) axis and sympatho-adrenomedullary (SAM) system (Yehuda et al., 1992; Liberzon et al., 1999; de Kloet et al., 2005; McEwen, 2005; Heim et al., 2018; Iob et al., 2020), which mediates homeostatic stress responses. Systemically-released adrenal stress hormones, cortisol (CORT; corticosterone in rodents) and epinephrine (E), are key elements in these pathways. During exposure to a stressor, the immediate SAM response is driven by the hypothalamus, which stimulates the sympathetic nervous system that then sends the signals to the adrenal medulla to release epinephrine. This “fight or flight” response is short-lived compared to HPA activity (Wong et al., 2012). Once the HPA axis is activated, homeostasis is maintained through negative feedback to prevent overstimulation. Circulating adrenal hormones reduce central secretion of their triggers such as corticotropin-releasing hormone (CRH) or adrenocorticotropic hormone (ACTH) (Ulrich-Lai and Herman, 2009). Disruption of these homeostatic loops and/or persistent SAM activation, often by prolonged stress, is commonly found in depression and post-traumatic stress disorder (PTSD) (Yehuda et al., 1991; de Kloet et al., 2005, 2006; Dwyer et al., 2020). The consequences of chronic stress exposure or HPA/SAM dysregulation are mixed, with some studies reporting hyper-responsivity (increased CORT release), and others reporting hypo-responsivity, or a blunted CORT response (Pitman and Orr, 1990; Nemeroff, 1996; Yehuda, 2006), or no difference (Klaassens et al., 2012). These inconsistencies may reflect individual differences in stress sensitivity and highlight the need for a more nuanced understanding of adrenal function under stress. Specifically, methods are needed for probing the capacity for adrenal release and feedback adaptation at multiple points along the stress and recovery trajectory.

Circulating adrenal hormones also affect learning. Memories formed during high emotional arousal can persist and be reactivated more efficiently than memories formed during low arousal (Cahill and McGaugh, 1998). These memory-enhancing effects can be adaptive or problematic, depending on the specific memory and its degree of generalization. For instance, trauma-related disorders involve formation of extinction-resistant emotional memories that lead to pathological avoidance habits (American Psychiatric Association [APA], 2013). There is great interest in finding ways to alter extinction learning processes to oppose these traumatic memories (Kida, 2019). Pre-clinical studies have suggested timed brain stimulation (Milad and Quirk, 2002; Vidal-Gonzalez et al., 2006; Bukalo et al., 2015; Likhtik and Paz, 2015), glutamatergic agonists (Norberg et al., 2008; La Buissonnière-Ariza et al., 2020), and timed increases in adrenal hormones (de Kloet et al., 2005, 2006; McEwen, 2005; Klaassens et al., 2012; Iob et al., 2020) as potential strategies for augmenting extinction. Invasive brain stimulation presents a challenge to routine clinical practice, and pharmacologic strategies have shown limited effectiveness in formal trials (Storch et al., 2007; Litz et al., 2012; Ressler, 2020). Part of the challenge is that manipulations may need to precisely coincide with the formation of extinction memories (Milad and Quirk, 2002). This is the basis of a recently-approved brain stimulation treatment for obsessive-compulsive disorder (Carmi et al., 2019). Methods for precisely timed adrenal release would enable pre-clinical studies of HPA and SAM effects on extinction learning.

**FIGURE 1:**
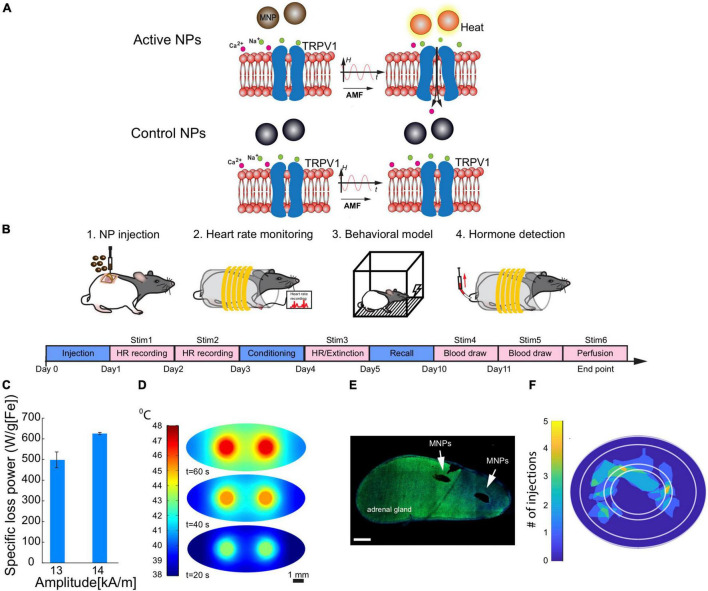
Experimental design. **(A)** When an alternating magnetic field (AMF) is applied, the magnetic nanoparticles dissipate heat that causes the opening of heat-sensitive calcium-permeable ion channels (TRPV1 receptors). The calcium influx causes E and CORT release from adrenal cells. **(B)** Schematic diagram of the procedures that were performed in order. 1. Adrenal MNP injections. 2. Heart rate monitoring before and during magnetothermal stimulation. 3. Aversive conditioning and extinction. 4. Serum collections during magnetothermal stimulation for hormone analyses. The experimental timeline was as follows: After adrenal MNP injections, heart rate monitoring took place during MNP stimulation for 2 days (days 1 and 2) before the 3-day behavioral testing (days 3–5). Five days later, blood was collected for serum hormone (E and CORT) measurements (days 10 and 11). **(C–F)** Adrenal injection specifications and histology. **(C)** The specific loss power (SLP) of MNPs at a frequency of 624 kHz was estimated for two amplitudes (13 and 14 kA/m) that were measured within the coil. **(D)** Finite element modeling of temperature increases at 2 small-volume MNP injection sites within the adrenal gland at 20-s, 40-s, and 60-s of AMF applied. Scale bar = 1 mm. **(E)** Representative mosaic of an adrenal gland section with two MNP injections (two black holes). Scale bar = 500 μm. **(F)** A map of MNP and WNP (control) injection sites in the adrenal gland across all animals (*n* = 16).

We recently demonstrated an approach for temporally precise adrenal hormone control: direct, wireless adrenal gland stimulation (Rosenfeld et al., 2020). Biocompatible, non-toxic magnetic nanoparticles (MNPs) composed of iron oxide can be injected into adrenal glands (or almost any peripheral organ). In humans or larger animal models, that injection could be performed under X-ray, magnetic resonance, or ultrasound guidance, i.e., as a minimally invasive procedure. In the presence of alternating magnetic fields (AMFs), the MNPs dissipate heat, opening endogenous thermosensitive calcium-permeable ion channels from the transient receptor potential (TRP) family and depolarizing electrogenic cells (Figure 1A). This approach increases circulating epinephrine and corticosterone in rats (Rosenfeld et al., 2020), controlled by calcium influx into adrenal cells. This technology offers advantages over other means of probing peripheral organ function. It permits access to tissues where chronic indwelling hardware (e.g., electrodes, catheters) may be difficult to implant or secure. It can be applied without a tether, implying potential use in multi-animal assays such as social interaction. Magnetic fields readily penetrate deep into tissue, and using magnetic nanomaterials as transducers enables spatially restricted stimulation. This contrasts with optical and ultrasonic approaches, where resolution and penetration depth are inversely correlated (Yizhar et al., 2011; Bystritsky and Korb, 2015; Niu et al., 2018; Christiansen et al., 2019; Fekete et al., 2020). Magnetic activation has advantages over other forms of hardware-free control such as chemogenetics, as it permits tight temporal control over organ stimulation (Shahriari et al., 2020).

As adrenal hormones have been shown to enhance extinction behavior and heart rate, we expected that using MNP technology to stimulate adrenal hormone release would elicit similar enhancements. In this study, we demonstrate this technology’s ability to probe HPA axis/SAM function at a specific timepoint and modulate behavior. We show that an acute stressor (threat conditioning) alters adrenal function and provide pilot evidence that MNP-activated timed adrenal hormone release can facilitate explorations of stress-related physiological and behavioral responses.

## Materials and Methods

### Nanoparticle Synthesis and Characterization

Iron oxide nanoparticles (NPs), either magnetic (active MNP) or non-magnetic wüstite (control NP) were synthesized by altering the solvent ratios during synthesis. First, we mixed sodium oleate (95%, TCI America) and 30 mmol of FeCl_3_⋅6H_2_O (99%, Acros Organics), heated to reflux (60°C) in a solvent mixture of hexane, ethanol and ddH_2_O for 1 h under N_2_. The mixture was heated to 110°C and dried overnight on a hotplate. MNPs were prepared by degassing the iron-oleate mixture at 90°C for 1 h in 2:1 (volume ratio) of 1-octadecene (90%, 10 ml) and dibenzyl ether (98%, 5 ml) and heated to 200°C under N2. Control NPs were prepared similarly but with 20 ml of 1-octadecene as solvent. Mixtures were then heated to 200°C under N_2_ and then to reflux at ∼325°C for 30 min. Both types of particles were 20–22 nm in diameter. They underwent surface functionalization using poly(ethylene glycol) grafted with poly(maleic anhydride-alt-1-octadecene) (Rosenfeld et al., 2020). NP concentration was measured using inductively coupled plasma-optical emission spectroscopy (ICP-OES,Agilent 5100 DVD), where *in vivo* injection concentration was ∼40 mg_[Fe]/_ml. For estimating the specific loss power (SLP, heating efficiency per gram of iron) of the NPs based on the dynamic hysteresis model (Christiansen et al., 2014), we used a similar calorimetry method described previously in our work (Chen et al., 2013). An optical fiber probe (Omega HHTFO-101) was used to measure temperature change in the ferrofluid solution inside a 7.5 mm gap of a toroidal ferromagnetic core that is driven by a custom-built series resonant circuit generating AMF with frequency *f* = 515 kHz and amplitude range of *H*_0_ = 13–15 kA/m. The SLP of MNPs in those conditions was estimated by 880 ± 38 W/g_[Fe]_ (mean ± standard deviation, std.) while the same conditions applied to control NPs yield a negligible SLP of 8 ± 4 W/g_[Fe]_ (Rosenfeld et al., 2020).

### Finite Element Modeling

To estimate the needed volume and concentration of MNPs and the number of injection sites for the *in vivo* experiments that will allow the MNPs to reach the threshold temperature of TRPV1 ion channel (42°C), we created a Finite Element Modeling (FEM) of heat distribution based on the gland dimensions, blood perfusion and fat layer surrounding the gland. The model was developed using Pennes’ bio-heat equation (Rosenfeld et al., 2020). We modified the previous model to fit the current experiment (Table 1).

**TABLE 1:** Parameters for finite element modeling (FEM).

Parameter	Value
Specific loss power of MNPs	600 W/g_[*Fe*]_
MNPs concentration	40 mg/ml
Blood density, ρ_*b*_	1,000 kg/m^3^
Heat capacity blood, C_*p,b*_	4,180 J/(kg⋅K)
Blood perfusion rate, ω_*b*_	0.0064 s^–1^
Arterial blood temperature, T_*b*_	37°C
Initial and boundary temperature, T_*o*_	37°C
Heat capacity adrenal, C_*p,A*_	3,540 J/(Kg⋅K)
Adrenal density, ρ_*A*_	1,020 kg/m^3^
Adrenal thermal conductivity, K_*A*_	0.52 W/(m⋅K)
Heat capacity fat, C_*p,F*_	2,348 J/(Kg⋅K)
Fat density, ρ_*F*_	911 Kg/m^3^
Fat thermal conductivity, K_*F*_	0.21 W/(m⋅K)

The chosen dimensions for the glands were modeled as an ellipsoid of 5.5 × 2.2 × 2.2 mm^3^ (with additional fat layer) and the following equations:


$$\rho_{\text{A}}{\text{C}}_{\text{A}}\frac{\partial T}{\partial t}={\text{K}}_{\text{A}}\nabla^{2}\text{T}+\rho_{\text{b}}{\text{C}}_{\text{b}}{\text{w}}_{\text{b}}\left(\text{T}-{\text{T}}_{\text{b}}\right)+\text{P}\text{A}\left\{\mathrm{adrenal}\mathrm{tissue}\right\}$$

$${\text{P}}_{\text{F}}{\text{C}}_{\text{F}}\frac{\partial T}{\partial t}={\text{K}}_{\text{A}}\nabla^{2}\text{T}+\rho_{\text{b}}{\text{C}}_{\text{b}}{\text{w}}_{\text{b}}\left(\text{T}-{\text{T}}_{\text{b}}\right)+\text{P}\;\text{F}\left\{\mathrm{fat}\mathrm{tissue}\right\}$$

### Subjects

We tested magnetothermal stimulation in male Long Evans rats weighing 250–300 g (Charles River, Wilmington, MA). The current study extends previous findings (Rosenfeld et al., 2020) by investigating this MNP technology’s ability to modulate behavior. Only males were used to maintain a comparable sample to that of the previous study. As sex differences have been reported (and reviewed) in aversive and extinction behaviors (Milad et al., 2009; Lebron-Milad et al., 2012; Maeng and Milad, 2015; Velasco et al., 2019), it will be critical that future studies examine whether MNP effects differ in females.

**FIGURE 2:**
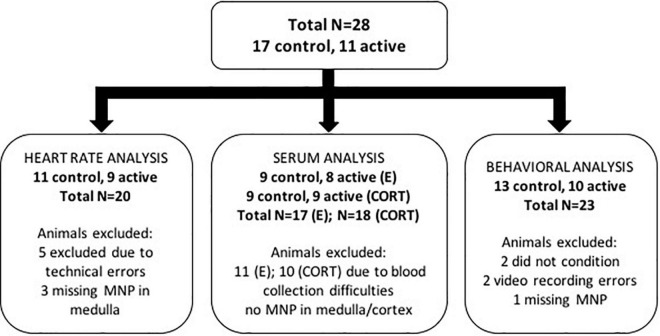
Summary chart of animal exclusions by analysis. A total of 28 rats were used in this study. Of these 28 animals, a number of animals were excluded from each of the heart rate, serum, and behavioral analyses for the reasons described in the diagram.

For this methodological proof of concept, we did not plan a fully-powered study but estimated the number of animals per group based on expected attrition. Rather, we planned for 20 animals per group (active MNP vs. control NP). Of these 40 initial rats, 28 completed the full protocol and were retained in at least some aspects of the analysis; Figure 2 diagrams the reasons for attrition per analysis. Rats were maintained on a 12 h light/dark cycle and ad libitum chow and water. Experiments were performed during the light phase of the light/dark cycle. All procedures were approved by the Subcommittee on Research Animal Care at the Massachusetts General Hospital (an Institutional Animal Care and Use Committee).

### Adrenal Magnetic Nanoparticle Injections

All rats were injected with intraperitoneal (i.p.) atropine (0.01 mg/kg) for respiratory support. About 20 min after injection, the rats were placed into an induction chamber and anesthetized with isoflurane. After induction, animals were injected with subcutaneous (s.c.) narcotic pain reliever buprenorphine (0.05 mg/kg), an anti-inflammatory non-narcotic pain reliever flunixin meglumine (2.5 mg/kg, s.c.), and intramuscular (i.m.) antibiotic enrofloxacin (5 mg/kg). To minimize the surgical burden each animal experienced, only the left adrenal gland was injected with MNPs or control NPs. The left adrenal gland was targeted because it is more easily accessible and has a larger medullary volume, with comparable secretory function to the right adrenal gland (De Nardi et al., 2017). The incision site was shaved and cleansed with alternating Betadine and ethanol. After the incision was made, the left adrenal gland was visualized and isolated (Rosenfeld et al., 2020). A Hamilton syringe containing nanoparticles was connected to a stereotaxic infusion pump and injected 1 μl (0.1 μl/min) into the gland in at least two different locations for a total volume of 2 μl, targeting both the adrenal medulla and cortex. The needle was kept inside the gland for 10 min post-injection to prevent leakage of the ferrofluid solution. Surgeries were performed by LYM, MFM, and DR. LYM and DR were aware of which injections contained control NP vs. MNP, but MFM was blinded. The data analysis was similarly performed blind to condition (see below). Rats recovered from surgery for at least 7 days before entering the experiment.

### Magnetothermal Stimulation

We measured HPA axis function by acute magnetothermal stimulation of awake, restrained rats (Figure 1B). Rats were placed inside a plastic restraint tube, to which they had all been habituated. The habituation procedure (multiple sessions of exposure to the tube and coil apparatuses) was important to minimize stress due to handling and the procedure used for the magnetothermal stimulation. The tube was then placed inside the core of an *in vivo* AMF stimulation apparatus, which was a replica of that used in Rosenfeld et al. (2020). The coil was driven by a custom resonant circuit and actively water cooled. The SLP (heating efficiency) of MNPs at the frequency of 624 kHz was estimated for two amplitudes that were measured within the coil (13–14 kA/m; Figure 1C) and were matched to reach an SLP of at least 600 W/g in MNPs as in the prior study (Rosenfeld et al., 2020). Control NPs under the same conditions yielded a negligible SLP (Rosenfeld et al., 2020). For all stimulation procedures, rats received 1 min of active stimulation (longer than the 40 s used in the previous study due to the rats’ larger/older status). Stimulation parameters were selected based on the amount of heat and its spread in the adrenal gland (Figure 1D), according to the FEM.

### Heart Rate Monitoring

To verify that adrenal hormone (particularly epinephrine) release occurred in response to MNP heating, we measured stimulation-induced heart rate changes. A pulse oximetry sensor was attached to the paw after each animal was restrained, and heart rate (bpm) was recorded before, during, and after stimulation using PhysioSuite (Kent Scientific, Torrington, CT). The experimenter performing stimulation and heart rate procedures (MFM) was blind to each rat’s experimental condition (MNP vs. control NP).

### Behavior

To assess the effects of adrenal stimulation on emotionally valenced learning, we employed a tone-shock conditioning/extinction/recall paradigm that we (Maeng et al., 2017b,a) and others (Quirk et al., 2000; Milad and Quirk, 2002) have previously used to test putative learning and memory enhancers. The experimenter (MFM) was blind to each rat’s experimental condition. Similar to prior experiments, behavior included habituation to the apparatus (to minimize novelty or stress effects in response to the environment), tone-shock conditioning, tone-only extinction, and tone-only extinction recall (Figure 1B). The behavioral testing procedure was run across 3 consecutive days. For each phase, an 82 dB tone conditioned stimulus (CS) was presented. On day 1, all rats underwent a habituation phase that consisted of 5 trials of tone CS-alone presentations. Habituation was immediately followed by the aversive conditioning phase. Conditioning consisted of 7 trials of tone CS and shock unconditioned stimulus (US) pairings. In the conditioning trials, each tone culminated with a 0.5 mA foot shock administered by the grid floor in the operant chamber. The extinction phase took place on day 2, consisting of 20 tone-CS alone trials. Immediately prior to extinction, each animal received 1 min of magnetothermal stimulation within the coil described above. Pre- and post-stimulation blood samples were collected 6 and 7 days after extinction. We quantified defensive behavior (freezing) by video analysis (ANY-maze, Stoelting Co., Wood Dale, IL). On day 3, the Recall phase comprised 3 CS-alone trials. During each phase, video recordings were analyzed to assess freezing behavior during the experimental trials. Freezing behavior was analyzed via ANY-maze (Stoelting Co., Wood Dale, IL, United States) post-experimentation using video recordings taken within the operant chamber during each behavioral testing phase (habituation, conditioning, extinction, and recall).

### Serum Hormone Quantification

Five days after behavioral testing, we further verified hormone release by lateral tail vein blood collections before and immediately after stimulation. To increase vasodilation of the lateral tail vein and thus blood flow, tails were warmed for 2–4 min with hand warmers. The animals were anesthetized with 0.25 mg/kg ketamine to insert a tail vein catheter, then allowed to recover for blood collection. After the catheter was implanted, it was flushed with saline and blocked with heparin (0.2 ml). Blood samples were drawn after removing the heparin block. After at least an hour of sitting at room temperature for the blood to clot, the samples were centrifuged at 5,000 rpm for 5 min at room temperature. We chose to perform this collection only after the conditioning and extinction paradigm because we had already verified pre-conditioning hormone release properties (Rosenfeld et al., 2020). Methodological limitations surrounding repeated blood sampling, such as the brief stimulation time window, unintended stress effects on subsequent behavioral measures, etc., did not allow us to perform repeated blood sample collections during the behavioral test phases. Serum hormone levels were quantified via ELISA (MyBioSource, Epinephrine: MBS264776, Corticosterone: MBS761865) and a plate reader to determine the optical density. For each tested hormone, the absolute values were calculated according to a calibration curve, obtained separately for each tested plate.

### Histology and Image Analysis of Adrenal Glands

Following the experiment, rats were sacrificed via injections of pentobarbital/phenytoin at 150 mg/kg (Beuthanasia-D C IIIN, Merck Animal Health, Patterson Veterinary, Devens, MA) and transcardial perfusion with 0.9% saline and 4% paraformaldehyde at a rate of about 21 ml/min. To confirm the nanoparticle injection locations, the adrenal glands in agarose were sectioned using a vibratome with a thickness of 40 μm, mounted on glass slides, and imaged in a laser scanning confocal microscope Fluoview FV1000, Olympus). The percentage of nanoparticle coverage of each adrenal sub-region was determined from mosaic scans of the entire adrenal slice via image analysis (Rosenfeld et al., 2020). Post-processing transformed each adrenal slice to an ellipse with the same semi-axes. This permitted labeling of adrenal sub-regions: medulla, zona glomerulosa (ZG), zona fasciculata (ZF) and zona reticularis (ZR). A map of injection locations was generated across 16 adrenal glands injected with NPs and defined the percentage of area covered with MNPs for each injected gland (Figures 1E,F).

### Statistical Analysis

All analyses were performed using RStudio (RStudio Team, 2020; R Core Team, 2021) with packages betareg (Cribari-Neto and Zeileis, 2010), broom (Robinson et al., 2021), fitdistrplus (Delignette-Muller and Dutang, 2015), ggplot2 (Wickham, 2016), ggpubr (Kassambara, 2020), imputeTS (Moritz and Bartz-Beielstein, 2017), plotrix (Lemon, 2006), readxl (Wickham and Bryan, 2019), and tidyverse (Wickham et al., 2019). As noted above, this study was not powered to detect significant between-group differences, and we present parametric statistics primarily to demonstrate possible effect sizes. The complete code for all analyses, with corresponding data files, is available online at https://github.com/tne-lab/magnex. For all analyses, we identified appropriate distributions for statistical testing by visualization of the Cullen-Frey graph in fitdistrplus (Delignette-Muller and Dutang, 2015).

#### Statistical Analysis – Heart Rate

We analyzed heart rate data before, during, and after stimulation for Days 1 and 2 (pre-conditioning) and Day 4 (post-conditioning) separately, retaining only animals with adequate data and verified NP placement. Due to animal movement and occasional loss of the pulse oximetry signal, heart rate tracings contained gaps. We imputed these missing values using Kalman smoothing (“imputeTS” package, “na_kalman” function), using the default parameters. Post-imputation, data were scaled by dividing each measurement in the time series by the mean pre-stimulation heart rate. Day 1 and 2 data were averaged at each time point for each respective animal. Day 4 data was left separate because measurements were obtained after the conditioning phase and thus were expected to have had different underlying adrenal biology. We retained only animals where we were able to successfully impute missing data. We excluded animals in the active MNP group if the histology showed no particles in the medulla. This left *n* = 11 control and *n* = 9 active rats for this analysis. We summed samples within the stimulation time range (300–360 s) to compute the area under the heart rate curve (AUC). These values best fit a gamma distribution, and thus we compared active and control conditions using a generalized linear model with gamma distribution, identity link function and a single independent variable (treatment condition).

#### Statistical Analysis – Serum

After excluding animals for experimental failures and outlier values, we analyzed the mean of the serum hormone levels across the 2 days of collection. We converted the data to a post-stim/pre-stim ratio, which we compared between active and control animals with a two-sample *t*-test on the log of the post/pre ratio. Serum analyses assessed epinephrine and corticosterone levels immediately prior to and after stimulation. 9 animals were excluded from analysis due to blood sampling issues, e.g., catheter malfunctions. For the remaining animals, the serum values were best described by a beta distribution. We then excluded animals with outlier pre-stimulation hormone levels, defined by fitting a beta distribution to all pre-stimulation hormone values (corticosterone and epinephrine separately). We excluded the serum samples whose baseline was extremely unlikely, i.e., *p* < 0.0005 based on the fitted distribution. This method excluded 1 additional animal. Finally, animals without active MNP placement in the medulla for epinephrine, or the cortex for corticosterone release, were also excluded (animals retained for E, control: *n* = 9, active: *n* = 8; CORT, control: *n* = 9, active: *n* = 9). We then converted the data to a post-stim/pre-stim ratio. These values were consistent with a log-normal distribution for each hormone, hence active and control animals were compared with a two-sample *t*-test on the log of the post/pre ratio.

#### Statistical Analysis – Behavior

We quantified freezing behavior across the 3-day paradigm as the percentage of the 30 s CS tone that was spent freezing. The behavioral paradigm consisted of four testing phases: habituation (day 1), conditioning (day 1), extinction (day 2), and recall (day 3). We analyzed freezing with a beta regression model, which is designed for data spanning the 0.00 ≤ x ≤ 1.00 interval. This beta regression analysis was selected because it was a more appropriate fit to the distribution of the data than a repeated measures ANOVA. For each animal, the raw freezing scores were adjusted to span this full range using a min-max normalization [(x - min)/(max - min)]. Normalizations were done separately for each phase of the testing procedure. These normalized data were scaled by dividing by each rat’s individual baseline freezing, defined as the average of the final 3 trials of the habituation phase (Trial 3–5). Finally, we smoothed the normalized trial-to-trial data using a centered moving average with window size 3. Post-normalization, we confirmed that freezing data fit a beta distribution. Data then were analyzed in a beta regression using trial, testing phase, and treatment (fixed effects, including 2-way and 3-way interaction terms) as explanatory variables. Before the analysis, we excluded animals that did not initially condition, and thus that could not have experienced extinction learning. Conditioning failures were determined by visual inspection of the conditioning behavior curve, by an investigator (ASW) who was blind to each rat’s treatment assignment. We also excluded animals in the active MNP group where both cortex and medulla were particle-free. These approaches excluded 5 rats (21.7% of total), with *n* = 23 rats retained in the analysis (control: *n* = 13; active: *n* = 10). For the analysis comparing the final three trials (Trials 18–20) of extinction, we confirmed that these averages followed a normal distribution before applying a *t*-test.

## Results

### Adrenal Magnetic Nanoparticle Injection Verification

Histological results verifying the location of the NP adrenal injections are illustrated in Figures 1E,F. Most of the injected NPs were found in the ZR, medulla, and ZF, while none of the NPs were found in the ZG (data tables available on GitHub).

### Magnetic Nanoparticle Influences on Heart Rate

**FIGURE 3:**
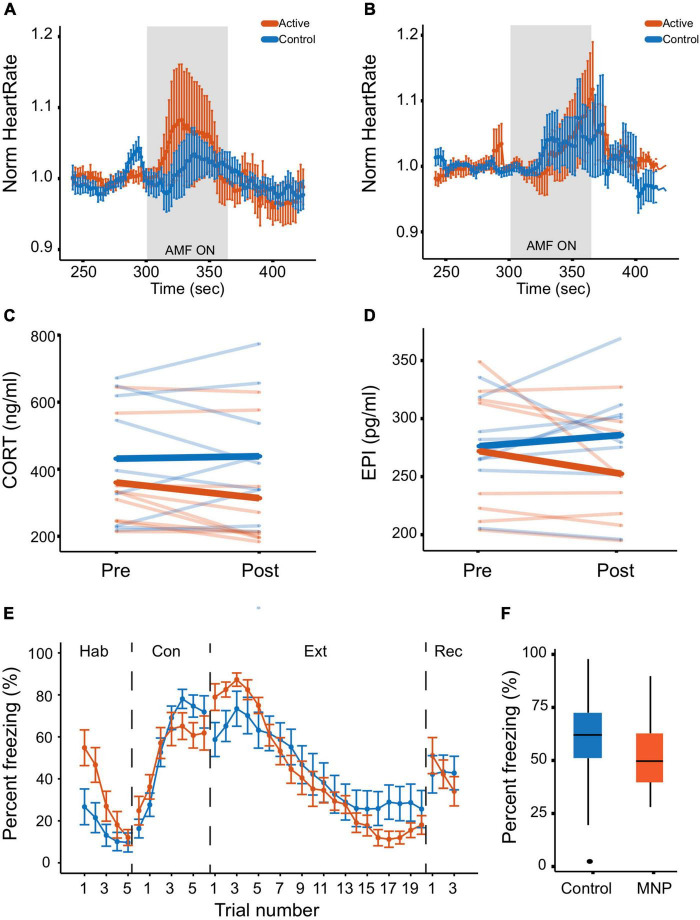
Magnetothermal adrenal stimulation effects on heart rate and hormone levels. **(A,B)** Heart rate measurement during magnetothermal stimulation on **(A)** days 1 and 2 before conditioning and **(B)** on day 4 after conditioning. **(C,D)** Serum hormone analysis. **(C)** Corticosterone and **(D)** epinephrine were measured before (pre) and after (post) magnetothermal stimulation. **(E,F)** Behavioral effects of magnetothermal adrenal stimulation. **(E)** Percent freezing per trial across the habituation, conditioning, extinction, and recall phases of the behavioral paradigm (controls, *n* = 13; active, *n* = 10). **(F)** Percent freezing averaged across the last three trials of extinction (Trials 18–20) for control and active MNP rats.

Prior to tone-shock conditioning, animals injected with active MNPs showed adrenal engagement in response to an AMF stimulus, evidenced by an increased heart rate during stimulation compared to controls (Figure 3A). The between-group difference did not reach significance in this small sample [*t*(14) = 0.807, *p* = 0.433]. Further, aversive conditioning changed adrenal responsivity. After conditioning (but before extinction on day 4), MNP stimulation produced similar changes in heart rate between the control and active MNP animals [*t*(14) = −0.025, *p* = 0.98] (Figure 3B). Heart rate rose more slowly in the animals injected with active MNPs, and there was greater variability as stimulation continued (increasing width of error bars specifically during the stimulation period).

### Magnetic Nanoparticle Modulation of Adrenal Hormones

After aversive conditioning and extinction, the circulating hormone response to magnetothermal stimulation was also altered. In animals injected with active MNPs, serum CORT and E both decreased from pre- to post-stimulation (Figures 3C,D). The change in CORT reached statistical significance compared to control MNPs [*t*(16) =−2.2, *p* = 0.047], whereas the change in E did not [*t*(14) = −1.8, *p* = 0.091], although significance should be evaluated with caution given the sample size. Importantly, these are the reverse of the change we previously reported in unconditioned animals (Rosenfeld et al., 2020).

### Magnetic Nanoparticle Modulation of Defensive Behavior

Freezing to conditioned tones did not significantly differ between the animals injected with active MNPs and those injected with control non-magnetic NPs in any experimental phase (Figure 3E). There was a non-significant enhancement of extinction learning from adrenal stimulation at the end of extinction [*t*(20.4) = −0.579, *p* = 0.569], that did not persist to the recall phase (Figure 3F).

## Discussion

We previously demonstrated remote control of adrenal hormone release via magnetically triggered heating of locally injected MNPs (Rosenfeld et al., 2020). Here, we demonstrated the use of this technology to probe the state dependence of HPA axis function. After aversive conditioning, this adrenal stimulation produced no visibly different changes in heart rate, contrary to the HR increases that were previously observed (Rosenfeld et al., 2020). This may represent exhaustion of a readily releasable pool if animals with continuously high adrenergic tone after conditioning were left with little reserve E available for release in response to subsequent stimulation (McCarty et al., 1988). This may offer a model for persistent hyper-arousal in trauma/stress-related disorders and demonstrates the potential role of this technology as a probe of neuro-endocrine interactions. MNP stimulation also decreased serum E and CORT, while in our prior study of non-conditioned animals, the same stimulation increased these hormones at the same time point (Rosenfeld et al., 2020). In addition to not being conditioned, another possible explanation for the hormone and HR differences between the two studies may be the anesthetized state. The animals in the previous study were under isoflurane anesthesia, compared to the awake and behaving state of the animals in the current study. Overall, the reduction in CORT after MNP stimulation aligns with the heart rate results, in that it again represents a blunted response compared to the previous study. These decreases potentially reflect an up-regulation of E/CORT metabolism or feedback inhibition as a result of a repeatedly activated HPA axis during conditioning, which could lead to a net decrease by the post-stimulation measurement (Kannan, 2012). These results further demonstrate that magnetothermally-driven adrenal release can probe aversive learning-related alterations of HPA and SAM function.

Behaviorally, we did not observe a significant difference in freezing, even though circulating adrenal mediators might be expected to improve extinction learning (Merz et al., 2014; Wolf et al., 2016; de Quervain et al., 2019). The present study observed a reduction in pre- to post-stimulation serum CORT levels, suggesting that the lack of a behavioral difference may be driven by a lack of sustained change in circulating mediators, or even by their suppression. The timing of activation/secretion may also be important. Previous studies suggest time-dependent hormone effects on behavior, e.g., hydrocortisone reduced defensive behavior only if given close to the time of training (Merz et al., 2018). Because our technology allows more precise control over hormone release timing compared to commonly used approaches such as a systemic injection or delivery via an osmotic pump, it could permit dissection of this time dependence.

The current approach was limited to adrenal gland activation by triggering heat-sensitive TRP channels, in a relatively non-specific way across the entire organ, in restrained animals. However, the resulting limitations can be overcome. For example, we have activated mechanosensitive ion channels by altering the MNP structure and magnetic field conditions (Gregurec et al., 2020). Moreover, magnetothermal stimulation can also inhibit cell activity by activating heat-sensitive hyperpolarizing channels (Munshi et al., 2018). To note, as biological tissues have negligible magnetic susceptibility in the frequency range of 100 kHz–10 MHz, we can achieve specific magnetothermal modulation at a frequency around 600 kHz without significant risk of general effects of the magnetic field. Controlled injections of MNPs to specific substructures within the adrenal gland, instead of the current multiple-injection approach, could increase specificity and permit the independent release of individual hormones such as androgens and aldosterone. We found a greater percentage of MNPs in the zona reticularis, but we did not measure its androgen hormone product, dehydroepiandrosterone (DHEA), which could be a consideration for future investigations. Similarly, the zona glomerulosa, which did not contain MNPs in this study, could be a target region for investigating the effects of aldosterone, another adrenal hormone implicated in stress responses and depression (Jezova et al., 2019; Izakova et al., 2020). Specificity might also be achieved using a multiplexing approach for controlling different populations of MNPs within the same organ (Moon et al., 2020). While we used restrained animals, the current coil design would permit experiments with freely moving mice (Rao et al., 2019). Freely moving rat experiments are possible, but demand scaling up of the coils and the driving power electronics (Christiansen et al., 2017).

The lack of female inclusion was a limitation of this study, but as mentioned above, deciding to use the same type of animals as in (Rosenfeld et al., 2020) was necessary as an extension of the previous findings. Inclusion of females in future MNP studies is critical as sex hormones such as estrogen, progesterone, and testosterone affect learning processes including extinction (Milad et al., 2010; Merz et al., 2012; Hwang et al., 2015; Hamson et al., 2016; Maeng et al., 2017b,a; Taxier et al., 2020) and are broadly implicated in psychiatrically relevant functions (Andreano and Cahill, 2009; Dwyer et al., 2020; Hwang et al., 2021). In addition to testing in females, this technology could also address the role of sex hormones by generalizing beyond the adrenal gland and including the gonads. Systemic hormone administration in animal studies relies on subcutaneous or intraperitoneal injections, which can be stressful and interfere with the sex hormone effects being investigated. TRP receptors are present in the ovaries, testes, and hypothalamus (Stein et al., 2004; Kunert-Keil et al., 2006; Surkin et al., 2020). While the thermal effects in this study are attributed mainly to TRPV1 activation, verified also in our previous study, other temperature-sensitive ion channels could be potentially activated, for example TRPV3 with a threshold of 31–39°C (Broad et al., 2016). Therefore, the hypothalamic-pituitary-gonadal (HPG) axis could be similarly probed by magnetothermal stimulation.

Beyond their use as probes of peripheral organ function, magnetic nanomaterials have been increasingly recognized as therapies and “theranostic” tools. They are being applied for treating cancer, diagnosing atherosclerosis, delivering drugs, etc. (Williams, 2017; Gul et al., 2019). However, their utility as tools for neuropsychiatric treatment and investigation is less defined. While intracranial or deep brain MNP stimulation has mostly been validated with motor behaviors (Kozielski et al., 2021), these particles may be a valuable tool for creating and/or manipulating models of neuropsychiatric disorders (Rao et al., 2019; Lu et al., 2020). For instance, MNP stimulation of the prelimbic cortex reduced immobility in the forced-swim test and increased sucrose consumption in stressed mice (Lu et al., 2020). Rao et al. (2019) demonstrated the use of MNPs for targeted drug delivery, where MNP-stimulated drug release increased dopamine-mediated social behavior. While these findings illustrate how MNP stimulation can be used to alter behavior via central modulation, the present study suggests a potential for modulation in the periphery, which may be more accessible and translatable.

Magnetothermal or magnetomechanical approaches are not universally useful. Chemogenetics will be simpler wherever temporal precision is not necessary. Very complex arenas will be difficult to instrument with coils for MNP activation, favoring more mature, tethered technologies such as optogenetics. The coils require specialized driving hardware and high-voltage supply lines that may not be available in all laboratory environments. It would similarly be hard to run many animals at once using this paradigm, and pharmacological/chemogenetic tools would be more suited if high throughput is needed. As nanomaterials, MNPs still need to be placed into organs of interest and may degrade, migrate, or be eliminated over time, unlike larger optogenetic fibers (Kolosnjaj-Tabi et al., 2016; Tsoi et al., 2016).

Despite the limitations described above, we have demonstrated the use of MNPs to remotely and more precisely detect and assess alterations within a peripheral stress system. While we observed effects of MNP stimulation on heart rate, serum hormone levels, and behavior that were not in the direction that we hypothesized, our findings did illustrate how MNP stimulation allowed us to identify specific changes in the functioning of a stress system that has been altered by aversive conditioning. Future research could further examine the timing of MNP peripheral stimulation effects on behavior, influences in the brain, and their potential role in the restoration of post-conditioning adrenal function. As the technology becomes more refined and widely available, these on-demand peripheral release approaches could provide valuable new tools for understanding and eventually altering the biology of mental illness beyond the brain.

## Data Availability Statement

The datasets presented in this study can be found in online repositories. The names of the repository/repositories and accession number(s) can be found in the article/supplementary material.

## Ethics Statement

The animal study was reviewed and approved by the Subcommittee on Research Animal Care at the Massachusetts General Hospital (an Institutional Animal Care and Use Committee).

## Author Contributions

ASW and PA conceived and designed the study. MM, DR, and LM ran the behavioral experiment and serum analyses. FK, AS, JM, and GV helped with magnetic stimulation and image analysis. AR and AW performed follow-up analyses. LM and DR wrote the first draft of the manuscript. LM, DR, ASW, and PA wrote sections of the manuscript. ASW and GS performed the statistical analyses. All authors approved the submitted version.

## Conflict of Interest

ASW, PA, and DR are inventors on a patent application for therapeutic uses of controlled adrenal release. The remaining authors declare that the research was conducted in the absence of any commercial or financial relationships that could be construed as a potential conflict of interest.

## Publisher’s Note

All claims expressed in this article are solely those of the authors and do not necessarily represent those of their affiliated organizations, or those of the publisher, the editors and the reviewers. Any product that may be evaluated in this article, or claim that may be made by its manufacturer, is not guaranteed or endorsed by the publisher.
